# Wavelet-enhanced boundary adaptation network for liver hemangioma segmentation in non-contrast CT

**DOI:** 10.3389/fonc.2025.1725514

**Published:** 2026-01-27

**Authors:** Bohao Zeng, Lei Zhang, Liling Peng, Wenming Cao, Xiaotao Fan, Xinfeng Sun, Xin Gao

**Affiliations:** 1 School of Economics and Management, Chongqing Jiaotong University, Chongqing, Nan’An, China; 2 School of Mathematics and Statistics, Chongqing Jiaotong University, Chongqing, Nan’An, China; 3 Department of PET/MR, Shanghai Universal Medical Imaging Diagnostic Center, Shanghai, Xu’Hui, China; 4 Radiology Department, Fengdu General Hospital, Chongqing, Feng’Du, China

**Keywords:** CT images, liver hemangioma segmentation, local attention mechanism, patient safety, transfer learning, wavelet transformation

## Abstract

Liver hemangioma segmentation in non-contrast CT images faces significant challenges due to the absence of contrast-enhanced features. This paper introduces WLAU-Net, a novel architecture integrating three key innovations for contrast agent free segmentation. First, our transfer learning framework pre-trains the encoder on venous phase CT images to capture discriminative tumor features, then transfers and freezes these learned weights when processing non-contrast phase data, effectively preventing domain shift. Second, we implement a wavelet transformation module using sym4 wavelet decomposition to split images into four frequency subbands (LL, LH, HL, HH). By selectively amplifying horizontal (HL) and vertical (LH) edge coefficients during reconstruction, we enhance tumor boundary delineation while preserving anatomical context. Third, a local attention mechanism with Gaussian-based adaptive weighting dynamically prioritizes low-intensity tumor regions over high-intensity areas, sharpening focus on subtle boundaries. Experimental results demonstrate WLAU-Net’s superiority with a 65.37% Dice score and 96.23% ACC, outperforming state-of-the-art methods including CS-UNet (64.50% Dice, 93.85% ACC) and Swin-UNet (62.34% Dice, 91.15% ACC). Ablation studies reveal critical contributions from each component: enabling all modules (transfer learning, Gaussian attention, and wavelet enhancement) achieves optimal performance, while removing the wavelet module reduces Dice by 1.16% (64.21%) and disabling both Gaussian and wavelet modules decreases ACC by 3.0% (93.24%). Compared to contrast-enhanced methods (92.1% ACC), our approach maintains competitive diagnostic accuracy (96.23% ACC) while eliminating allergic risks, offering a clinically viable alternative for contrast agent sensitive patients.

## 1 Introduction

Liver hemangioma Segmentation in Computed Tomography (CT) plays a crucial role in diagnosis, surgical planning, and treatment monitoring. While contrast-enhanced CT (CECT) provides clear tumor boundaries through the injection of contrast agents, these agents pose significant risks for patients with allergies or renal insufficiency (1). Non-contrast CT, despite its safety, presents substantial segmentation challenges due to low soft-tissue contrast and ambiguous tumor boundaries (2).

In the field of medical image segmentation, the U-Net model, with its unique symmetric encoder-decoder architecture and skip connections to enhance detail preservation (3), has become the preferred choice for many researchers. Building upon this framework, significant advancements have been achieved in various medical tumor segmentation tasks, including brain tumor segmentation in magnetic resonance (MR) imaging, liver hemangioma segmentation using CT scans, and pancreatic tumor segmentation (4, 5).

While convolutional neural networks (CNNs) excel in feature representation, their limited ability to capture tumor edge features prompted the integration of attention mechanisms into U-Net, leading to the development of Attention UNet (6). Despite U-Net’s strong segmentation capabilities, the spatial limitations of convolutional operations restrict their global feature modeling. Recognizing this, researchers have increasingly turned to Transformers, which rely entirely on attention mechanisms and inherently excel at capturing global context (7). However, as Transformers focus more on global context modeling, hybrid approaches combining CNNs with Transformer encoders show greater potential. TransUNet, introduced in 2021, became one of the first models to apply Transformer technology to medical image analysis (8). This method leverages the U-Net encoder’s strength in capturing high-resolution spatial details while harnessing the Transformer’s ability to model global context, inspiring extensive follow-up research (9). Nevertheless, when applying TransUNet to non-contrast images, the lack of contrast enhancement results in indistinct tumor features and blurred edges, leading to discontinuous segmentation. Additionally, global attention mechanisms still struggle to precisely localize tumor regions (10, 11).

**Figure 1:**
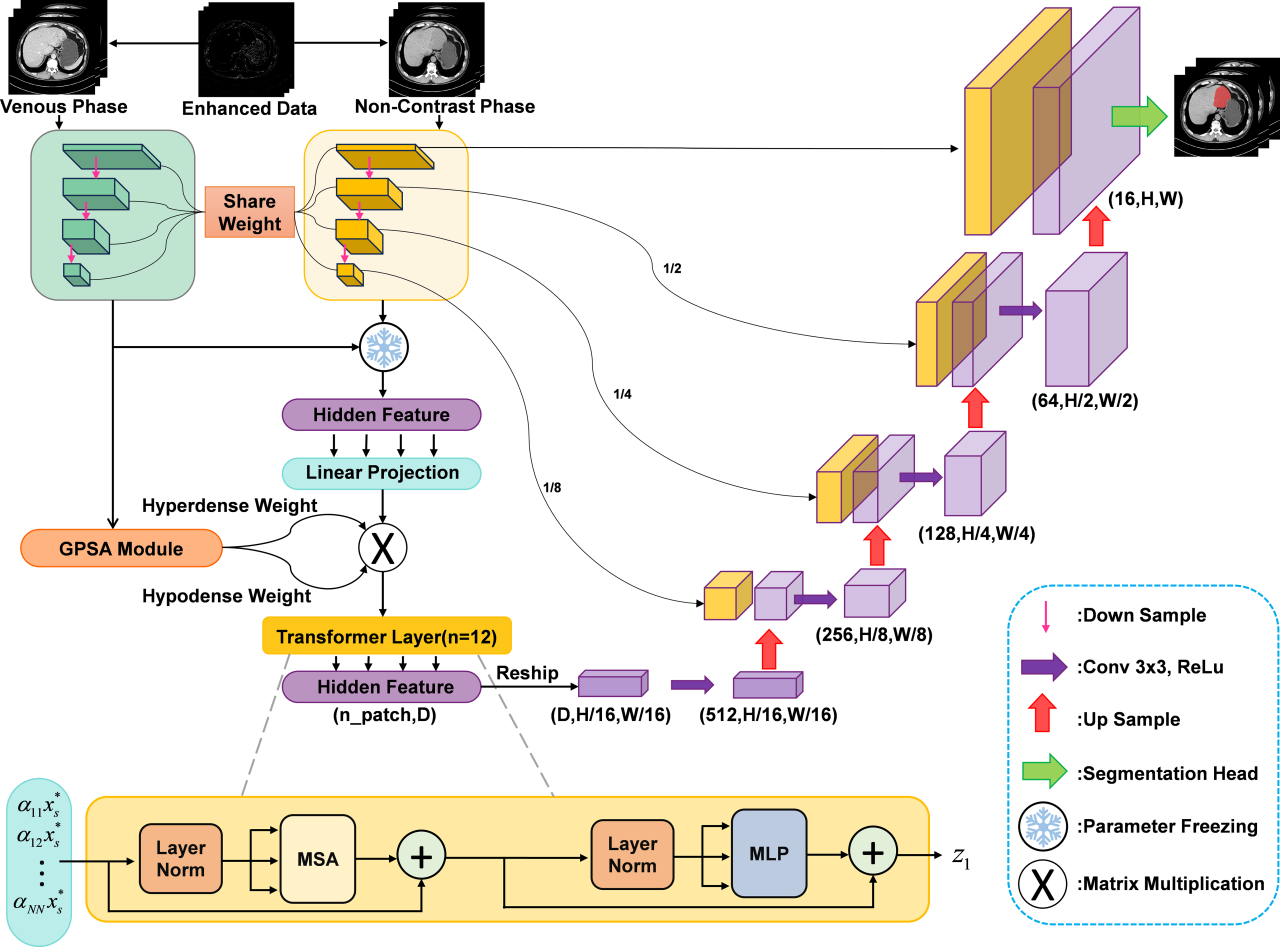
The overview of WLAU-Net framework.

To address these challenges, we propose the Wavelet and Local Attention UNet (WLAU-Net), a framework extending TransUNet for segmenting tumors in non-contrast CT images. First, the original non-contrast and venous phase data are input into a Wavelet-based Edge Enhancement Module (WEEM). This module decomposes the image into multi-frequency sub-bands (LL, LH, HL, HH), amplifies high-frequency components (HL and LH) via a frequency band residual amplification strategy, and reconstructs the image through inverse wavelet transform (12–14). By evaluating different wavelet functions and amplification coefficients, the optimal configuration is selected to enhance tumor edge features (15). Next, venous phase data are input with frozen CNN encoder parameters, while non-contrast data are used for training. We then introduce a Gaussian-based Position-sensitive Attention (GPSA) module, which dynamically weights CNN features using Gaussian functions (16). The GPSA-generated dynamically weighted feature maps are tokenized into patches and processed by a Transformer encoder. This enables seamless fusion of global self-attention features with high-resolution CNN features via skip connections for precise localization. Finally, the Transformer decoder reformulates pixel-wise segmentation into a mask classification task, treating predicted candidate regions as learnable queries (17). These queries interact with local multi-scale CNN features through a collaborative cross-attention mechanism, progressively refining the final results (overall framework in **Figure 1**).

In summary, the key contributions of this work are:

1. WLAU-Net, a novel hybrid architecture based on the TransUNet framework. Under a supervised cross-domain protocol, the model integrates wavelet transforms and Gaussian-based dynamic weighting to achieve precise segmentation of non-contrast liver hemangiomas in CT.
2. GPSA, a Gaussian-based dynamic weighting module addressing feature discrepancies between non-contrast and venous phase data in transfer learning. It effectively mitigates challenges in focusing on tumor regions in non-contrast images.
3. WEEM, an image reconstruction module enhancing tumor boundary features while preserving anatomical integrity. By decomposing images into frequency sub-bands and amplifying specific components via inverse wavelet transform, it significantly improves tumor edge recognition.
4. The proposed model enables accurate tumor segmentation in CT images without contrast agents, reducing allergy risks associated with their use.

The remainder of this paper is organized as follows: Section 2 describes the dataset, preprocessing methods, and reviews the TransUNet model while detailing the principles of WEEM and GPSA. Section 3 presents experimental setups, evaluates the proposed method using Dice Similarity Coefficient (Dice) and Hausdorff Distance (HD), and analyzes segmentation accuracy across tumors of varying sizes. Section 4 concludes the paper and outlines future research directions.

## 2 Materials and methods

### 2.1 Data preparation

In the segmentation of liver hemangiomas, the imaging differences between venous phase and non-contrast phase scans are crucial for accurate detection and analysis (18). Given the characteristic variations in hemangioma presentation across different imaging phases, precise differentiation and segmentation of these vascular lesions hold significant importance for subsequent diagnosis and treatment. Our research therefore focuses on feature transfer and automated segmentation tasks between venous phase and non-contrast phase imaging to enhance clinical diagnostic accuracy and efficiency. This study utilizes the private FDLT liver hemangioma dataset (approved by the Institutional Review Board, IRB No. 2024SC1015-1), comprising non-contrast and venous phase (contrast-enhanced) CT scans from 654 patients. All tumor regions were independently annotated by nine radiologists using ITK-SNAP software (version 4.2.0).

The abdominal CT scans were acquired using a SOMATOM Definition AS+ CT scanner (SIEMENS, Germany) at FengDu General Hospital. The imaging parameters were configured as follows: tube voltage = 120 kV; tube current = 52 mA; exposure time = 500 ms; slice thickness = 1.5 mm; reconstruction matrix size = 512 × 512; pixel spacing = 0.75 × 0.75 mm². The scan protocol included a venous phase enhancement (Series Description: Venous Phase 1.5 I30f) with a spiral pitch factor of 0.6 and a total collimation width of 38.4 mm.

Raw CT images were reconstructed using a convolution kernel of I30f, with a field of view (FOV) diameter of 384 mm. Spatial consistency was ensured through alignment with the patient coordinate system (Image Orientation: [1, 0, 0, 0, 1, 0]). To minimize radiation exposure, the volumetric CT dose index (CTDIvol) was optimized to 2.88 mGy, achieving a dose saving of 56.53%.

All CT images were normalized to Hounsfield Units (HU) and clipped to the range [-150, 250] HU to focus on liver tissue. Intensity values were then scaled to [0, 1]. Images were resampled to a uniform voxel spacing of 0.75 × 0.75 × 1.5 mm³ using linear interpolation. During training, data augmentation included random rotation (± 15°), scaling (0.9–1.1), and horizontal flipping (p=0.5).

Patients were included if they had at least one radiologically confirmed liver hemangioma visible in both non-contrast and venous phase scans. Exclusion criteria included: (1) previous hepatic surgery or intervention, (2) severe motion artifacts in CT images, and (3) concurrent malignant liver tumors.

The patient cohort consisted of 342 males (52.3%) and 312 females (47.7%), with an overall average age of 52.3 ± 12.7 years (range: 18–86 years). A total of 812 hemangiomas were manually annotated across all patients. The tumor size distribution, measured by the maximum diameter on the venous phase scans, exhibited considerable variation: diameters ranged from 3.2 mm to 87.5 mm, with a mean diameter of 24.6 ± 18.3 mm. Based on the maximum diameter, tumors were categorized into three groups: Tiny (*<* 10 mm, n=198, 24.4%), Small ([10, 20) mm, n=245, 30.2%), and Big (≥ 20 mm, n=369, 45.4%). Anatomically, the tumors were distributed across the liver as follows: left lobe (38.2%), right lobe (56.7%), and caudate lobe (5.1%).

The dataset was randomly split at the patient level into training (524 patients, 80%), validation (65 patients, 10%), and test sets (65 patients, 10%). This ensured no data leakage between sets.

All scans were performed with the patient in a head-first supine position (HFS), and reconstructed images were archived in compliance with anonymization protocols, retaining only non-identifiable demographic information. Due to ethical restrictions and patient data privacy concerns, the original datasets used in this study are not publicly available. However, in the interest of research transparency and reproducibility, the complete source code and models have been made publicly accessible at: https://github.com/Jinchengwu318/WLAU-net.

### 2.2 Related works

#### 2.2.1 Tumor feature migration and frequency-domain analysis

The domain shift between contrast-enhanced and non-contrast CT imaging poses significant challenges for cross-phase lesion analysis. Due to the absence of contrast agents, non-contrast images exhibit blurred tumor boundaries and attenuated texture features, leading to performance degradation when models trained on venous phase data are directly applied to non-contrast images (19). To address this, feature migration methods aim to transfer discriminative features between imaging phases while preserving critical tumor characteristics (20). Early approaches like CycleGAN attempted cross-phase synthesis but often failed to preserve anatomical structures and subtle lesion details (21, 22). More recently, frequency-domain adaptation has emerged as a promising direction. LUCIDA introduced Fourier-domain processing to align low-dose and full-dose CT data, demonstrating the potential of frequency manipulation for domain adaptation (23). However, Fourier transforms (FFT) provide global frequency representations that may not adequately capture multi-scale local structures in medical images (24). In contrast, discrete wavelet transform (DWT) offers localized time-frequency decomposition, simultaneously capturing global anatomical contours and local details such as tumor edge microtextures (15, 25, 26). By adjusting wavelet coefficients in specific sub-bands (LL, LH, HL, HH), DWT enables targeted enhancement of relevant features while maintaining robustness (27, 28). Building upon these insights, we incorporate DWT-based edge enhancement to address the domain gap in liver hemangioma segmentation.

#### 2.2.2 Transformer-based medical image segmentation

Transformers, originally developed for natural language processing (7), have revolutionized computer vision through their powerful self-attention mechanisms. In medical image analysis, Vision Transformer (ViT) demonstrated that global self-attention applied directly to image patches could achieve state-of-the-art performance (29). For segmentation tasks, TransUNet pioneered the integration of Transformers with UNet architectures, establishing a hybrid framework that combines CNN’s local feature extraction with Transformer’s global context modeling (8). Subsequent developments, including Swin-UNet (30), further advanced transformer-based segmentation through hierarchical feature processing. While these methods leverage global attention mechanisms, they typically lack domain-specific adaptations for cross-phase medical imaging. Our work extends this line of research by incorporating Gaussian-based positional attention guided by anatomical priors, specifically designed to address the challenges of non-contrast liver hemangioma segmentation.

### 2.3 Methods

Given a source domain dataset FDLT (*X_s_,V_s_*), where *X_s_* and *V_s_* denote the non-contrast phase images and their corresponding annotated venous phase images, respectively. Each image *x* ∈ *X_s_* is represented as R*^H^*^×^*^W^*^×^*^C^*, where *H*×*W* is the spatial resolution and *C* is the number of channels. Our objective is to accurately segment non-contrast phase data *X_s_* in the target domain (FDLT dataset) without contrast agent interference, while preserving the original spatial resolution *H* × *W*. A straightforward approach involves training a CNN to encode images into feature representations and subsequently decode them back to full resolution (3).

Unlike conventional CNN-based methods, our framework enhances the TransUNet architecture by integrating three key components: (1) wavelet transform for tumor boundary enhancement, (2) cross-phase transfer learning to bridge domain discrepancies, and (3) Gaussian-based reweighting of tumor feature map attention. The detailed implementations of these components are systematically elaborated in Sections 2.3.1, 2.3.2, and 2.3.3, respectively.

**Figure 1** illustrates the overall architecture of the proposed WLAU-Net. The model takes paired non-contrast phase *X_s_* and venous phase *V_s_*, CT images of size 512 × 512 as input. Both phases are first processed independently by the WEEM to accentuate the boundary features of liver hemangiomas. Subsequently, the enhanced images are fed into a 4-layer CNNs for feature extraction. Each CNN layer employs a 3 × 3 kernel with a stride of 2. The non-contrast branch produces a feature map *I_ij_*, which is then directed to the GPSA. The GPSA learns a spatial attention weight matrix *α_ij_* that guides the model’s focus toward regions likely to contain liver hemangiomas. Simultaneously, the venous phase branch, after passing through the same 4-layer CNNs, yields a feature map of size 32 × 32. This feature map undergoes a linear projection to transform it into a structure compatible with the Transformer encoder. The projected features are then modulated by the attention weights *α_ij_* before being input to the Transformer. The Transformer encoder comprises *n* = 12 layers, each utilizing multi-head self-attention with 8 heads. The output of the Transformer, denoted as *z*, is reshaped back into a 32 × 32 feature map. This map is progressively upsampled to the original resolution through a decoder pathway. Each upsampling stage uses bilinear interpolation with a 2×2 kernel, doubling the spatial dimensions per stage. The upsampled features are concatenated with the corresponding feature maps from the non-contrast CNNs encoder via skip connections. Each combined feature set is then processed by a convolution block, kernel size 3×3, stride 1, activated by a ReLU function. This decode-and-merge process is repeated four times. Finally, a segmentation head processes the refined high-resolution features to produce the final pixel-wise segmentation mask for liver hemangiomas in the non-contrast CT image.

#### 2.3.1 Wavelet-based edge enhancement module

**Figure 2:**
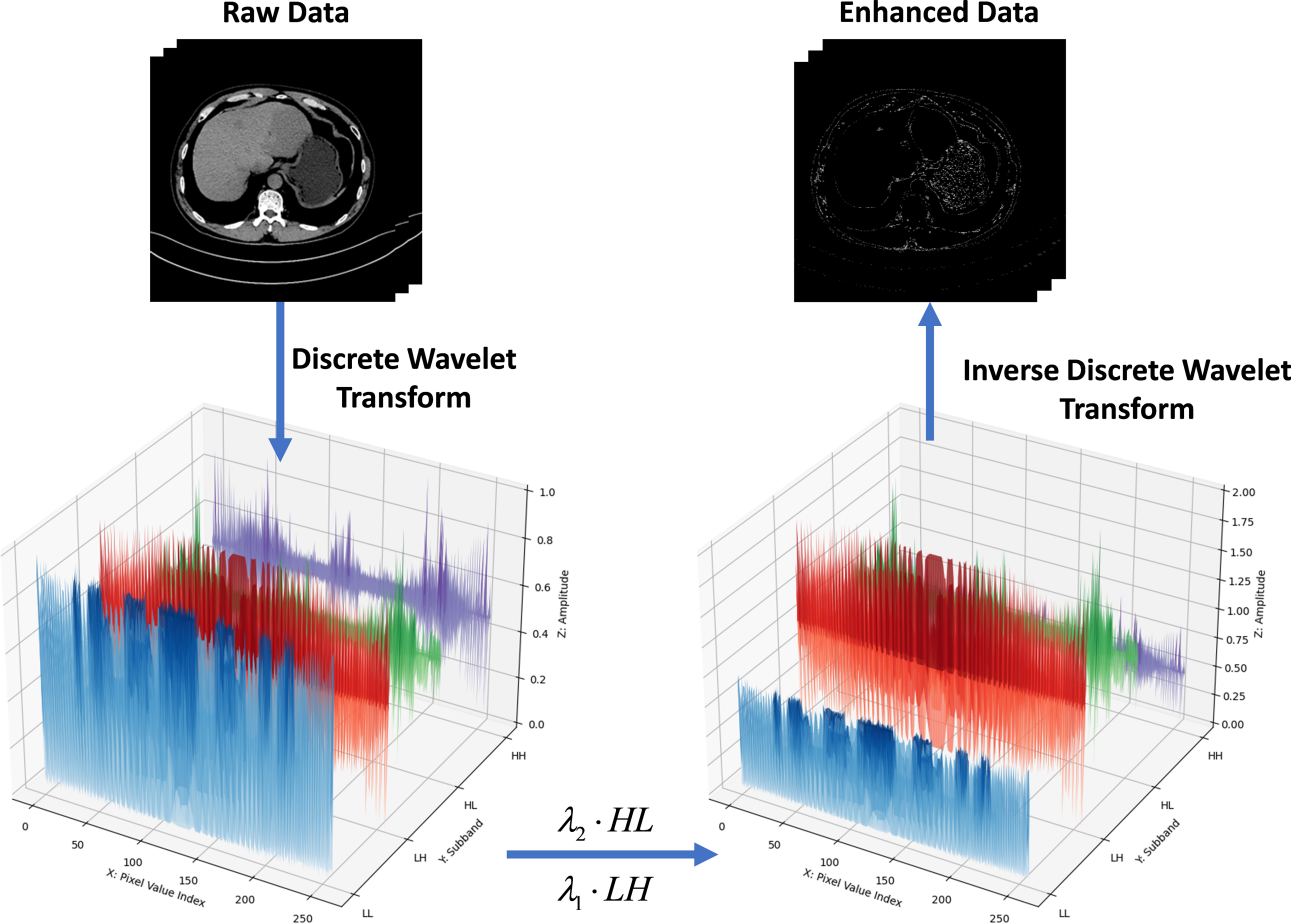
Performing wavelet transform, the amplitude of the HL/LH bands is doubled to enhance the tumor boundary features.

**Figure 2** introduces a *Sym4 wavelet decomposition module* to process both noncontrast phase (*X_s_*) and venous phase (*V_s_*) CT images from the FDLT dataset through 2D Sym4 wavelet transform, generating four distinct subbands (31). By strategically amplifying the amplitude coefficients in the HL and LH subbands prior to reconstruction, we enhance tumor boundaries without distorting anatomical structures.

The image reconstructed based on sym4 wavelet is defined as in Equation 1:

(1) $$\left\{ \begin{array}{l} W_{LL}(x_{s}),W_{LH}(x_{s}),W_{HL}(x_{s}),W_{HH}(x_{s})=\text{DWT}(x_{s})\\ W_{LL}^{*},W_{LH}^{*},W_{HL}^{*},W_{HH}^{*}=U_{\text{dwt}}(W_{LL},W_{LH},W_{HL},W_{HH}|\lambda )\\ x_{s}^{*}=\text{IDWT}(W_{LL}^{*},W_{LH}^{*},W_{HL}^{*},W_{HH}^{*}) \end{array}\right.$$

Where *x_s_* denotes an image sample from the source domain *X_s_*, the signal is decomposed via the Discrete Wavelet Transform (DWT) into four frequency subbands: *W_LL_* (low-frequency approximation), *W_LH_* (horizontal details), *W_HL_* (vertical details), and *W_HH_* (diagonal details). These subbands are optimized via the update function $U_{\text{dwt}}(\cdot |\lambda )$, where *λ* is a hyperparameter balancing feature enhancement and noise suppression, and *U*_dwt_ denotes a domain-adaptive parameterized operator. The refined subbands $W_{LL}^{*},W_{LH}^{*},W_{HL}^{*},W_{HH}^{*}$ are reconstructed into the enhanced signal $x_{s}^{*}$ through the Inverse Discrete Wavelet Transform (IDWT), where $x_{s}^{*}$ is the output signal preserving structural coherence and amplified boundary features, ensuring compatibility with downstream tasks such as segmentation or classification.

#### 2.3.2 Cross-phase transfer learning to bridge domain discrepancies

To effectively bridge the domain gap between contrast-enhanced and non-contrast CT images, we implement a structured pre-train, freeze, and transferstrategy. This approach begins by training the model encoder on venous phase data, where liver hemangiomas exhibit pronounced contrast enhancement and well-defined boundaries, thereby providing a rich and discriminative feature space for learning fundamental tumor characteristics. The choice of the portal venous phase as the source domain is motivated by its status as the clinical gold standard for liver lesion characterization, ensuring that the model acquires robust prior knowledge of hemangioma morphology and texture.

The encoder is optimized on the venous phase data by minimizing the Dice loss function *L*_Dice_ (Equations 2, 3):

(2) $$L_{\text{Dice}}=1-\frac{2|Y_{s}\cap \hat{Y}|}{\left|Y_{s}|+|\hat{Y}\right|}$$

(3) $$\theta^{*}=\text{arg }\underset{\theta }{\min}(1-\frac{2\sum_{v}Y_{s}(v)\hat{Y}(x^{*};\theta )}{\sum_{v}Y_{s}(v)+\sum_{v}\hat{Y}(x^{*};\theta )})$$

where *Y_s_* denotes the ground truth mask for the venous phase, $\hat{Y}$ is the predicted segmentation, and $\theta^{*}\;$represents the optimized encoder parameters. These parameters are subsequently frozen ( $\theta =\theta^{*}$) and directly transferred to initialize the model for the non-contrast phase segmentation task.

The decision to freeze the encoder parameters, rather than employing full fine-tuning, serves as a strategic regularization mechanism to prevent overfitting to the limited and low-contrast non-contrast data. By preserving the encoder’s learned representations, we ensure that the model retains the robust, domain-invariant features acquired from the source domain, thereby stabilizing the learning process in the target domain. This approach effectively decouples feature learning from domain adaptation: the frozen encoder provides a consistent, high-level representation of hepatic anatomy and tumor morphology, while the subsequent learnable modules—specifically the Gaussian-based Position-Sensitive Attention (GPSA) and the transformer decoder—are tasked with adapting to the nuances of non-contrast imaging. These modules learn to attend to and reconstruct the subtler tumor boundaries and textures within the stable feature space provided by the encoder, thereby directly mitigating the domain shift caused by the absence of contrast agent. This parameter-sharing strategy not only enhances computational efficiency by eliminating the need to retrain the encoder from scratch but also ensures consistent feature representation across imaging domains, ultimately improving the model’s ability to segment hemangiomas in non-contrast CT scans (32, 33).

#### 2.3.3 Gaussian-based position-sensitive attention with ground truth guidance

The input image $x^{*}$ is processed by the CNN to extract hierarchical features, producing feature map *I_ij_*. Leveraging the available tumor annotation masks *Y_s_*, a Gaussian weighting mechanism is applied to guide the model’s focus toward the tumor region with anatomical precision (34).

The ground truth-guided Gaussian weighting is defined as (Equations 4–6):

(4) $$\mu_{Y_{s}}=\frac{1}{\left|Y_{s}\right|}\sum_{(i,j)\in Y_{s}}I_{ij}$$

(5) $$\sigma_{Y_{s}}=\sqrt{\frac{1}{\left|Y_{s}\right|}\sum_{(i,j)\in Y_{s}}{\left(I_{ij}-\mu_{Y_{s}}\right)}^{2}+\in }$$

(6) $$G_{ij}=\text{exp }(-\frac{{\left(I_{ij}-\mu_{Y_{s}}\right)}^{2}}{2\sigma_{Y_{s}}^{2}+\in }),\;\in =10^{-5}$$

where *Y_s_* represents the ground truth tumor mask, |*Y_s_*| denotes the number of pixels within the tumor region, $\mu_{Y_{s}}$ is the mean intensity of tumor pixels, and $\sigma_{Y_{s}}\;$is the standard deviation characterizing the intensity distribution within the annotated tumor area.

For enhanced spatial sensitivity, a multi-scale Gaussian formulation is employed (Equations 7, 8):

(7) $$G_{ij}^{\left(k\right)}=\text{exp }(-\frac{{\left(I_{ij}-\mu_{Y_{s}}\right)}^{2}}{2{\left(k\cdot \sigma_{Y_{s}}\right)}^{2}+\in }),\;k\in \{0.5,\;1.0,\;2.0\}$$

(8) $$G_{ij}=\frac{1}{3}\sum_{k}G_{ij}^{\left(k\right)}$$

The Gaussian weights are integrated into the transformer architecture through a bias matrix mechanism (Equations 9–12):

(9) $$g=\text{Flatten}(G)\in {\mathbb{R}}^{N\times 1}$$

(10) $$B_{\text{gaussian}}=g\cdot g^{T}\in {\mathbb{R}}^{N\times N}$$

(11) $$A=\frac{QK^{T}}{\sqrt{d_{k}}}+\lambda B_{\text{gaussian}}$$

(12) Output=softmax(A)V

where *Q, K, V* are query, key, and value matrices derived from the feature map, *d_k_* is the key dimension, and *λ* is a learnable parameter initialized at 0.5.

To ensure the attention mechanism aligns with clinical annotations, a dedicated attention supervision loss is introduced (Equations 13, 14):

(13) $$L_{\text{attention}}=\frac{1}{N}\sum_{i=1}^{N}\text{BCE}(\text{softmax}(A_{i}),Y_{s,i})$$

(14) $$L_{\text{total}}=L_{\text{Dice}}+\alpha L_{\text{attention}}$$

where BCE denotes binary cross-entropy loss, and *α* controls the relative weight of attention supervision (empirically set to 0.3).

During inference, when ground truth annotations are unavailable, the model utilizes population-level statistics learned during training (Equations 15, 16):

(15) $$\mu_{\text{learned}}=\frac{1}{\left|D_{\text{train}}\right|}\sum_{n=1}^{\left|D_{\text{train}}\right|}\mu_{Y_{s}}^{\left(n\right)}$$

(16) $$\sigma_{\text{learned}}=\frac{1}{\left|D_{\text{train}}\right|}\sum_{n=1}^{\left|D_{\text{train}}\right|}\sigma_{Y_{s}}^{\left(n\right)}$$

This approach ensures that the Gaussian attention mechanism benefits from precise anatomical guidance during training while maintaining practical applicability during clinical deployment.

This ground truth-guided Gaussian attention mechanism provides strong anatomical priors that significantly enhance the model’s ability to focus on liver hemangiomas, particularly in non-contrast CT where lesion conspicuity is reduced (7).

#### 2.3.4 Hyperparameter tuning

To ensure optimal model performance, we conducted systematic hyperparameter tuning for WLAU-Net. The optimization process involved both manual exploration based on empirical evidence and automated search techniques, with performance evaluated on the validation set (10% of patients). The key hyperparameters and their configurations are summarized below.

##### 2.3.4.1 Training hyperparameters

We utilized the AdamW optimizer with an initial learning rate of 3 × 10^−4^, which was decayed using a cosine annealing schedule over 200 epochs. The learning rate search space spanned from 10^−5^ to 10^−3^. The batch size was set to 8, selected after testing values of 4, 8, and 16, with 8 providing the best trade-off between memory usage and gradient stability. Weight decay was applied with a coefficient of 10^−2^ to mitigate overfitting. For the transformer component, we used 12 layers with 8 attention heads, a configuration that consistently outperformed alternatives in preliminary experiments.

##### 2.3.4.2 Wavelet enhancement parameters

For the Wavelet-based Edge Enhancement Module (WEEM), we selected the Symlet-4 (sym4) wavelet based on its superior performance in boundary preservation, as evidenced by comparative analysis across multiple wavelet families (see **Table 1**). The enhancement coefficients for the horizontal *λ*_1_ and vertical *λ*_2_ detail subbands were optimized through grid search within the range [1.3, 2.2] with a step size of 0.1. The optimal values were determined to be *λ*_1_ = 2.0 and *λ*_2_ = 2.0, which provided the best trade-off between edge enhancement and noise amplification, as validated by the highest Dice score on the validation set. The low-frequency LL and diagonal HH subbands were left unmodified to preserve anatomical integrity and suppress high-frequency noise, respectively.

**Table 1:** The best performance of different wavelet functions in liver hemangioma segmentation on the FDLT dataset.

Daubechies	Dice	Symlets	Dice	Biorthogonal	Dice	Coiflets	Dice
db1	53.21	sym2	54.67	bior1.1	52.44	coif1	53.22
db2	58.76	sym4	65.37	bior2.2	60.15	coif2	59.73
db3	55.9	sym6	59.12	bior2.4	58.9	coif3	62.1
db4	61.33	sym8	57.45	bior3.1	55.67	coif4	56.32
db5	50.89	sym10	51.88	bior3.3	61.8	coif5	63.75

##### 2.3.4.3 GPSA module parameters

In the Gaussian-based Position-Sensitive Attention (GPSA) module, the scaling factor *λ* for the Gaussian bias matrix was initialized at 0.5 and made learnable during training. The attention supervision weight *α* in the total loss function was set to 0.3 after experimenting with values from 0.1 to 1.0. Multi-scale Gaussian kernels with standard deviation factors *k* ∈ {0.5, 1.0, 2.0} were empirically chosen to capture both local and global contextual information.

##### 2.3.4.4 Optimization process

The hyperparameter tuning process followed a staged approach: (1) initial coarse search for learning rate and batch size, (2) systematic evaluation of wavelet functions and enhancement coefficients, and (3) fine-tuning of attention-related parameters. The final configuration was determined based on the model’s performance on the validation set, measured primarily by the Dice Similarity Coefficient. This comprehensive tuning strategy ensured that WLAU-Net achieved robust and reproducible segmentation performance across varying tumor sizes and imaging characteristics.

## 3 Results

**Table 2** shows the results of liver hemangioma segmentation experiments based on the FDLT dataset, demonstrating that WLAU-Net outperforms several key metrics. As shown in **Table 2**, our method leads all comparison models with a Dice coefficient of 65.37%, an IoU of 58.53%, and an accuracy (ACC) of 96.23%. The Dice score improves by 0.87 percentage points over the next best model, CS-UNet (64.50%), while the IoU and ACC increase by 5.72 and 2.38 percentage points, respectively, compared to Swin-UNet (52.81%) and CS-UNet (93.85%). Although the Hausdorff Distance (HD) of 23.55mm is slightly higher than that of CS-UNet (22.37mm), it still outperforms mainstream architectures such as UNet (30.83mm) and TransUNet (27.62mm). This result indicates that our method effectively balances global anatomical integrity and local detail accuracy in liver hemangioma segmentation.

**Table 2:** Experimental results of the FDLT dataset. The Dice coefficient (Dice), Intersection over Union (IoU), Accuracy (Acc), and Hausdorff Distance (HD) for liver hemangioma segmentation across compared methods are presented.

Method	Dice↑	HD↓	IoU↑	ACC↑
UNet (3)	58.15	30.83	48.32	87.63
DeepLabV3+ (35)	59.83	29.47	49.75	88.91
Attention-UNet (6)	62.10	26.89	51.12	91.23
TransUNet (8)	61.45	27.62	50.68	90.27
Swin-UNet (30)	62.34	25.89	52.81	91.15
CS-UNet (36)	64.50	22.37	57.23	93.85
Ours	65.37	23.55	58.53	96.23

**Table 3** shows the impact of different module combinations in WLAU-Net on liver hemangioma segmentation performance. With the full configuration (T-T-T), the model achieves the best performance across all metrics (Dice of 65.37, HD of 23.55, IoU of 58.53, and ACC of 96.23). When wavelet transform, Gaussian module, or transfer learning module are sequentially removed, the model performance decreases to varying extents. For example, with the T-TF configuration, the Dice drops to 64.21 and the HD increases to 24.31; with the T-F-T configuration, the HD rises to 25.83 and the ACC drops to 94.88; with the F-T-T configuration, the Dice is 62.47 and the ACC drops to 93.57. In more extreme combinations, such as T-F-F, F-T-F, F-F-T, and F-F-F, all metrics show significant degradation. Among these, the HD is highest at 28.93 in the F-F-T configuration, and the IoU is lowest at 50.68 and ACC lowest at 90.27 in the F-F-F configuration. These results validate the effectiveness of each module in improving the overall performance of the model.

**Table 3:** Ablation study on module components for liver hemangioma segmentation (FDLT dataset). Performance is compared across different combinations of transfer learning, GPSA, and WEEM modules. Evaluation metrics include Dice, HD, IoU, and ACC.

Transfer	GPSA	WEEM	Dice↑	HD↓	IoU↑	ACC↑
			61.45	27.62	50.68	90.27
		✓	61.93	28.93	52.76	91.03
	✓		61.72	27.97	51.52	91.53
✓			62.53	27.48	53.90	93.24
	✓	✓	62.47	26.12	55.83	93.57
✓		✓	63.70	25.83	57.16	94.88
✓	✓		64.21	24.31	56.87	95.42
✓	✓	✓	65.37	23.55	58.53	96.23

**Figure 3:**
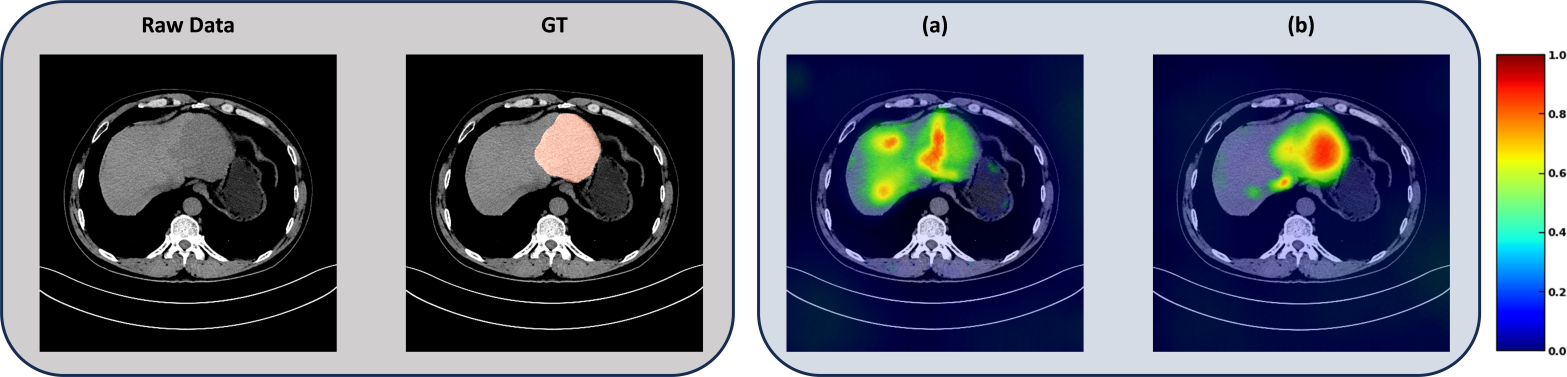
Attention visualization brought by the GPSA module: **(a)** shows the effect of disabling the Gaussian module, and **(b)** shows the effect of enabling the Gaussian module.

**Figure 3** shows the attention visualization brought by the GPSA module in the ablation experiment. (a) is the attention distribution of the model without the Gaussian module, and (b) is the attention distribution after adding the GPSA module.

**Figure 4:**
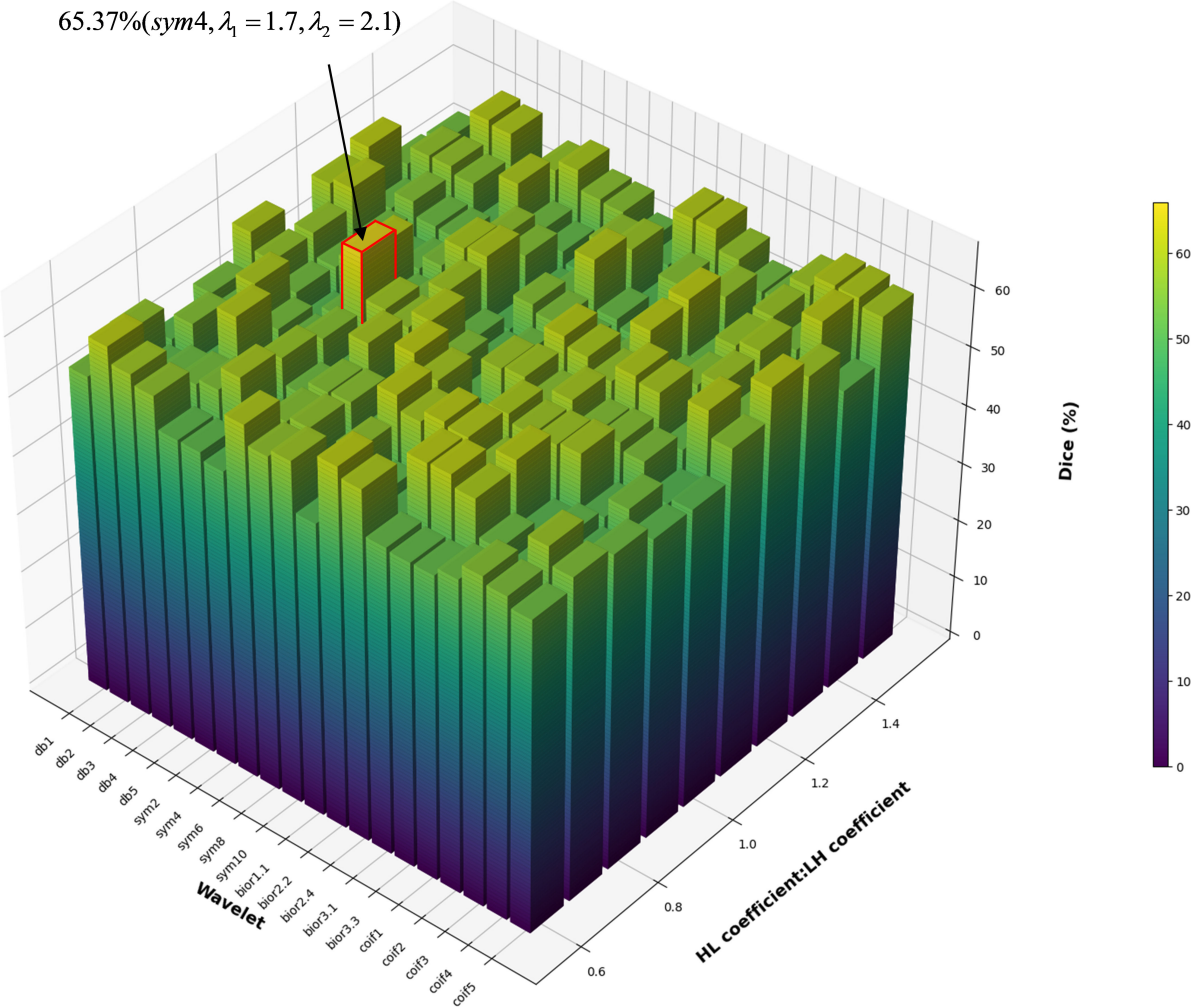
Dice values generated by different wavelet functions and different subband coefficients.

**Figure 4** shows the segmentation results of three cases, comparing our method with the baseline method TransUNet and other methods. Our method performs excellently in cases 1 and 2, segmenting medium and large liver hemangiomas. However, in case 3, all methods show partial segmentation errors for multiple small liver hemangiomas.

**Table 4** shows the significant performance differences of the methods in detecting liver hemangiomas of different sizes. Our method (Ours) demonstrates high accuracy in detecting liver hemangiomas of all sizes, with particularly outstanding performance in detecting small (Small) and large (Big) liver hemangiomas. For Tiny (*<*10 mm) liver hemangiomas, CS-UNet (54.57%) and our model (Ours, 52.15%) perform the best, significantly outperforming other methods (36). For Small ([10, 20) mm) liver hemangiomas, our method (Ours, 61.45%) achieves the best performance, followed by CS-UNet (61.30%) and TransUNet (60.37%) (8, 36). In the detection of Big (≥20 mm) liver hemangiomas, our method (Ours, 69.22%) also stands out, with Swin-UNet (67.21%) and CS-UNet (67.08%) following closely behind (30, 36). Overall, our method achieves the best detection accuracy in liver hemangiomas of various sizes, especially in Small and Big liver hemangiomas, demonstrating strong precision and robustness. It is suitable for clinical detection of liver hemangiomas and can provide more accurate diagnostic results.

**Table 4:** Tumor segmentation performance (average Dice) under different tumor sizes reported in the FDLT dataset.

Method	Tiny *<* 10 mm	Small [10, 20) mm	Big ≥ 20 mm
UNet (3)	49.86	55.21	62.03
DeepLabV3+ (35)	50.52	56.84	63.21
Attention-UNet (6)	51.21	58.45	62.50
TransUNet (8)	50.91	60.37	63.85
Swin-UNet (30)	51.88	59.15	67.21
CS-UNet (36)	54.57	61.30	67.08
Ours	52.15	61.45	69.22

**Table 1** shows that different wavelet functions perform differently in liver hemangioma segmentation. Among the Daubechies wavelets (such as db4, db6, db8, etc.), db4 performs the best with a Dice value of 58.76. Among the Symlets functions, the sym4 wavelet performs the best with a Dice value of 65.37. Among the Biorthogonal wavelets (such as bior2.2, bior3.3, etc.), bior3.3 stands out with a Dice value of 61.8. Among the Coiflets wavelets, the coif5 wavelet performs the best with a Dice value of 63.75. Overall, Symlets and Biorthogonal wavelet functions perform better in segmentation accuracy.

**Figure 4** shows the Dice effects produced by different inverse transform parameters corresponding to various wavelet functions, where the red-bordered ones represent the best wavelet function and inverse transform subband coefficients.

**Table 5** provides a detailed architectural comparison of the evaluated segmentation methods for liver hemangioma segmentation. As illustrated, WLAU-Net represents the most comprehensive framework, uniquely integrating all six key architectural components: CNNs, Transformer-based self-attention, attention mechanisms, cross-phase transfer learning, wavelet-based frequency-domain processing. In contrast, existing methods typically incorporate only a subset of these design elements. For instance, UNet and DeepLabV3+ rely solely on CNN-based architectures, while Swin-UNet employs a pure Transformer design. Attention-UNet and CS-UNet enhance CNN frameworks with attention mechanisms, and TransUNet combines CNN and Transformer components. However, only WLAU-Net incorporates the specialized modules—cross-phase transfer learning, WEEM, and GPSA—that are specifically designed to address the challenges of liver hemangioma segmentation in non-contrast CT, including domain shift between imaging phases and enhancement of subtle tumor boundaries. This holistic architectural design enables WLAU-Net to achieve superior performance, as demonstrated by its leading segmentation metrics in **Table 2**.

**Table 5:** Architectural comparison of liver hemangioma segmentation methods.

Method	CNNs	Transformer	Attention	TL	Wavelet
UNet (3)	✓				
DeepLabV3+ (35)	✓		ASPP		
Attention-UNet (6)	✓		✓		
TransUNet (8)	✓	✓	✓		
Swin-UNet (30)		✓	✓		
CS-UNet (36)	✓		✓		
WLAU-Net (Ours)	✓	✓	✓	✓	✓

## 4 Discussion

The WLAU-Net framework proposed in this study demonstrates significant advancements in non-contrast CT liver hemangioma segmentation. By innovatively integrating wavelet transform and attention mechanisms, it successfully addresses the inherent challenges of insufficient contrast in non-enhanced imaging. Experimental results reveal that the model outperforms existing mainstream methods with a Dice score of 65.37% and accuracy of 96.23%, validating the synergistic effectiveness of frequency-adaptive enhancement and dynamic attention mechanisms. Notably, the wavelet edge enhancement module significantly improves tumor boundary recognition through selective amplification of high-frequency components, while the Gaussian-weighted local attention mechanism effectively suppresses interference signals, enabling precise capture of subtle tumor features. Compared with conventional contrast-enhanced approaches, this solution achieves superior accuracy while completely avoiding contrast agent risks, offering a safer clinical alternative.

**Figure 5:**
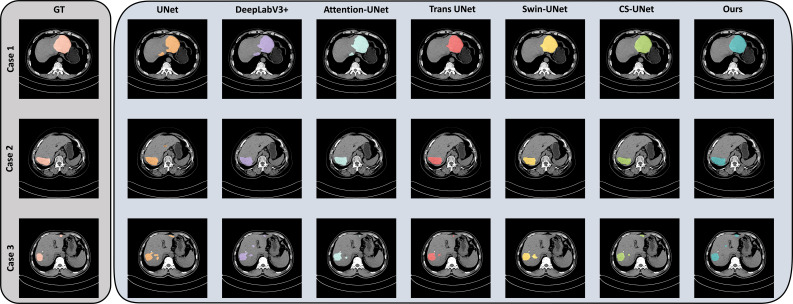
Visualization of segmentation results of different methods in the three test cases.

Segmentation Error Analysis and Implications for Real-time Application. To assess the model’s clinical applicability, we conducted a detailed analysis of segmentation errors, particularly focusing on patterns that may impact real-time deployment. As illustrated in **Figure 5** (Case 3), the model exhibits a tendency to under-segment clustered small hemangiomas. This error pattern likely stems from the limited receptive field of local attention mechanisms when confronted with multiple small lesions in close proximity, where the boundaries between adjacent tumors become ambiguous in non-contrast imaging. Furthermore, boundary uncertainty persists in low-contrast tumors where intensity gradients are subtle, occasionally leading to either over- or under-estimation of lesion extent. These error patterns have direct implications for real-time clinical application: first, they highlight the need for robust post-processing algorithms to refine boundary delineation and handle lesion multiplicity; second, they underscore the trade-off between processing speed and segmentation accuracy. Real-time intraoperative applications may require further optimization to meet stricter latency constraints. Future iterations could explore adaptive computation pathways that allocate more resources to challenging cases (e.g., clustered small tumors) while streamlining processing for straightforward ones, thereby balancing accuracy with speed.

Despite these breakthroughs, several limitations require improvement. First, the single-center data source may constrain model generalizability, necessitating future multi-center validation. Second, computational efficiency needs optimization, particularly regarding increased inference time from combining wavelet transform with TransUNet. Additionally, optimal frequency combinations may require anatomical site-specific adjustments, warranting further investigation. To translate WLAU-Net from a promising prototype into a clinically robust tool, our future work will prioritize two pivotal directions. First, to address the single-center limitation and ensure generalizability, we plan to conduct largescale, multi-center external validation. Collaborating with diverse medical institutions will allow us to test the model on data from varied CT scanners, imaging protocols, and patient demographics, which is essential for establishing its reliability in real-world clinical settings. Second, to tackle the computational overhead introduced by the wavelet and Transformer modules, we will focus on model lightweighting and deployment optimization. Techniques such as neural architecture search, knowledge distillation, or the design of efficient hybrid attention blocks will be explored to significantly reduce parameter count and inference latency without compromising accuracy. This optimization is crucial for practical integration into hospital picture archiving and communication systems (PACS) or potential edge-device deployment. By advancing in these directions, WLAU-Net can evolve into a practical, efficient, and widely applicable solution for contrast-free liver hemangioma assessment, directly addressing the needs of contrast-agent-sensitive patients. Overall, this research pioneers new pathways for contrast-free medical image analysis, with technical approaches extendable to other segmentation tasks like brain tumor delineation.

## 5 Conclusions

This study innovatively proposes WLAU-Net, a deep learning framework specifically designed for non-contrast CT liver hemangioma segmentation. By organically combining three key technologies - wavelet transform, transfer learning, and attention mechanisms - it effectively resolves core challenges of feature scarcity and boundary ambiguity in non-enhanced medical image analysis. Experimental evidence confirms that the model not only enhances segmentation accuracy but more importantly eliminates contrast-induced allergic risks, providing safe diagnostic solutions for vulnerable patient populations.

The theoretical contribution lies in establishing a frequency-adaptive medical image analysis methodology, while practical significance resides in developing clinically applicable intelligent diagnostic tools. The theoretical contribution of this work lies in establishing a frequency-adaptive analysis methodology for medical images, while its practical significance is embodied in the development of an intelligent diagnostic tool with clear clinical relevance. To bridge the gap between technical achievement and widespread clinical adoption, immediate future efforts will be dedicated to rigorous multi-center validation to confirm the model’s robustness across diverse populations and hardware, and to systematic model lightweighting to enhance its efficiency for real-time clinical use. Furthermore, we will explore the extensibility of the core methodology—integrating wavelet-based enhancement and guided attention—to other challenging contrast-free segmentation tasks, such as the delineation of brain tumors or renal lesions. These steps are crucial for advancing the field of agent free medical imaging and for ultimately providing safer, accessible diagnostic options for all patients.

## Data Availability

Access to the original dataset is restricted to protect patient privacy, in compliance with the ethical approval (Fengdu General Hospital IRB No. 2024SC1015-1). De-identified data are available from the corresponding authors upon reasonable request and with permission from the institutional ethics committee. Requests to access the datasets should be directed to XS, kanyisheng@163.com.
